# Complete mitochondrial genome of *Pterodecta felderi* (Lepidoptera: Callidulidae)

**DOI:** 10.1080/23802359.2020.1833777

**Published:** 2020-11-11

**Authors:** Keon Hee Lee, Min Jee Kim, Jeong Sun Park, Iksoo Kim

**Affiliations:** a Department of Applied Biology, College of Agriculture & Life Sciences, Chonnam National University, Gwangju, Republic of Korea; b Herbal Medicine Resources Research Center, Korea Institute of Oriental Medicine, Naju, Republic of Korea

**Keywords:** Mitochondrial genome, *Pterodecta felderi*, Callidulidae, Phylogeny

## Abstract

We report the mitochondrial genome (mitogenome) of *Pterodecta felderi* (Callidulidae: Lepidoptera), which is the first mitogenome sequences in the family Callidulidae, a monotypic family in the superfamily Calliduloidea. The 15,340-bp long complete mitogenome consists of a typical set of genes (13 protein-coding genes [PCGs], 2 rRNA genes, and 22 tRNA genes) and 1 major non-coding A + T-rich region, which are arranged in a way that is frequently observed in Lepidoptera. Of the 13 PCGs, 12 *P. felderi* start with ATN, except for COI, which starts with CGA. The *P. felderi* mitogenome consists of 210-bp long intergenic-spacer sequences and 27-bp long overlaps. Phylogenetic analysis of superfamilial relationships in the lepidopteran clade Obtectomera with concatenated sequences of the 13 PCGs and 2 rRNA genes using the Bayesian inference method showed that Calliduloidea, which is only represented by *P. felderi*, was placed as the most basal lineage about Macroheterocera (Lasiocampoidea, Bombycoidea, Mimallonoidea, Noctuoidea, and Drepanoidea), Papilionoidea, and Pyraloidea.

*Pterodecta felderi* Bremer, 1864 belongs to the monotypic family Callidulidae in the lepidopteran superfamily Calliduloidea. The species is distributed in Korea, southeastern Siberia, China, Taiwan, and Japan, and is found casually in mountainous regions of Korea (Shin 2001). Adults fly during the daytime in the spring and summer, and, at first glance, the shape and color appear to be similar to that of *Libythea lepita*, which belongs to the subfamily Libytheinae (snout butterflies) of the lepidopteran family Nymphalidae (Shin 2001). Up to now, no mitochondrial genome (mitogenome) of Callidulidae has been reported; despite the family is one of the essential taxa for phylogenetic reconstruction in Lepidoptera. Thus, in this study, we sequenced the complete mitogenome of the *P. felderi* that would be helpful for subsequent lepidopteran phylogeny by adding previously unavailable lepidopteran family.

Two legs of *P. felderi* adult were obtained from the tissue stocks preserved in Genetic Resources Bank of the National Institute of Biological Resources (NIBR), Ministry of Environment (MOE) of the Republic of Korea (NIBRGR0000073718). The collection information is as follows: Hwaya Mountain, Seorak-myeon, Gapyeong-gun, Gyeonggi-do Province, South Korea (37°41′22.4′′ N, 127°24′26.6′′ E). DNA was extracted using the Wizard Genomic DNA Purification Kit, in accordance with the manufacturer’s instructions (Promega, Madison, WI), and the remaining DNA was stored at Chonnam National University, Gwangju, Korea, under the accession number CNU12053. Using Lepidoptera-specific primers (Kim et al. 2012), three overlapping long fragments (LFs; COI to ND4, ND5 to lrRNA, and lrRNA to COI) were amplified. These LFs were then used as templates for the amplification of 26 short fragments using Lepidoptera-specific primers (Kim et al. 2012). Sequencing was performed for both strands by Sanger’s method.

Phylogenetic analysis using the nucleotide sequences of 13 protein-coding genes (PCGs) and two rRNA genes (12,514 bp) was performed for 10 species of Obtectomera including *P. felderi*, along with one species from Gelechioidea that was used as an outgroup (*Mesophleps albilinella*). Bayesian inference (BI) method implemented in CIPRES Portal version 3.1 (Miller et al. 2010) was conducted using MrBayes version 3.2.2 (Ronquist et al. 2012). An optimal substitution model (GTR + G, GTR + I+G, TVM + I+G) was determined using PartitionFinder 2 and Greedy algorithm (Lanfear et al. 2012, 2014, 2016).

The *P. felderi* mitogenome is 15,340 bp in length, with typical sets of genes (2 rRNA genes, 22 tRNA genes, and 13 PCGs) and a 400-bp long major non-coding A + T-rich region (GenBank accession number MT370823). The gene arrangement of *P. felderi* is identical to that of most lepidopteran species, and consists of the order tRNA^Met^-tRNA^Ile^-tRNA^Gln^ between the A + T-rich region and ND2 (Kim et al. 2010). This arrangement is different from that found in the ancient lepidopteran superfamilies like Hepialoidea (Timmermans et al. 2014) and Nepticuloidea (Cao et al. 2012) and from that of the ancestral arrangement found in the majority of insects (Boore 1999). The overall A/T nucleotide composition of the *P. felderi* mitogenome was as follows: 79.33% in the whole genome, 77.51% in PCGs, 84.61% in srRNA, 83.72% in lrRNA, 80.98 in tRNAs, and 95.50% in the A + T-rich region. Of the 13 PCGs, 12 *P. felderi* start with ATN, except for COI, which starts with the alternative CGA codon, as observed in other lepidopteran insects (Kim et al. 2012).

The phylogenetic tree showed that Calliduloidea is located as the most basal group about Macroheterocera (Lasiocampoidea, Bombycoidea, Mimallonoidea, Noctuoidea, and Drepanoidea), Papilionoidea, and Pyraloidea (Figure 1). Similar results have been obtained in studies by Cho et al. (2011), Mutanen et al. (2010), Regier et al. (2013), Heikkilä et al. (2015), and Breinholt et al. (2018); however, taxon diversity in this study is far limiting. Before this study, no complete mitogenome sequences of Callidulidae were available. Thus, more complete mitogenome sequences of the family would be required for further analysis of the relationships of the family and superfamily with other taxonomic groups.

**Figure 1.**
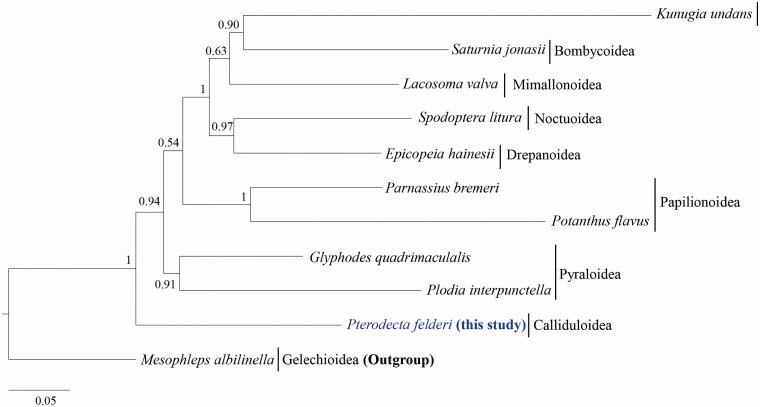
Phylogenetic tree of Obtectomera. Bayesian inference (BI) method was used for phylogenetic analysis based on the concatenated 13 PCGs and 2 rRNA genes (12,514 bp) using three partition schemes. The numbers at node indicate the Bayesian posterior probabilities (BPPs) determined by the BI method. The scale bar indicates the number of substitutions per site. *Mesophleps albilinella* belonging to Gelechiidae (KU366707; Park et al. 2016) was utilized as the outgroup. GenBank accession numbers are as follows: *Pterodecta felderi*, MT370823 (This study); *Spodoptera litura*, JQ647918 (Wan et al. 2013); *Saturnia jonasii*, MF346379 (Kim et al. 2018); *Kunugia undans*, KX822016 (Kim et al. 2017); *Glyphodes quadrimaculalis*, KF234079 (Park et al. 2015); *Plodia interpunctella*, KP729178 (Liu et al. 2016); *Parnassius bremeri*, FJ871125 (Kim et al. 2009); *Potanthus flavus*, KJ629167 (Kim et al. 2014); *Lacosoma valva*, KJ508050 (Timmermans et al. 2014); and *Epicopeia hainesii*, MK033610 (Yang et al. 2019).

## Data Availability

Mitogenome data supporting this study are openly available in GenBank at: https://www.ncbi.nlm.nih.gov/nuccore/MT370823. The data that support the findings of this study are openly available in Mendeley Data at http://dx.doi.org/10.17632/mdfnrrcvv2.1

## References

[CIT0001] Boore JL. 1999. Animal mitochondrial genomes. Nucleic Acids Res. 27(8):1767–1780. 10101183 10.1093/nar/27.8.1767 PMC148383

[CIT0002] Breinholt JW, Earl C, Lemmon AR, Lemmon EM, Xiao L, Kawahara AY. 2018. Resolving relationships among the megadiverse butterflies and moths with a novel pipeline for anchored phylogenomics. Syst Biol. 67(1):78–93. 28472519 10.1093/sysbio/syx048

[CIT0003] Cao YQ, Ma C, Chen JY, Yang DR. 2012. The complete mitochondrial genomes of two ghost moths, *Thitarodes renzhiensis* and *Thitarodes yunnanensis*: the ancestral gene arrangement in Lepidoptera. BMC Genom. 13:276. 10.1186/1471-2164-13-276 PMC3463433 22726496

[CIT0004] Cho S, Zwick A, Regier JC, Mitter C, Cummings MP, Yao J, Du Z, Zhao H, Kawahara AY, Weller S, et al. 2011. Can deliberately incomplete gene sample augmentation improve a phylogeny estimate for the advanced moths and butterflies (Hexapoda: Lepidoptera)? Syst Biol. 60(6):782–796. 21840842 10.1093/sysbio/syr079 PMC3193767

[CIT0005] Heikkilä M, Mutanen M, Wahlberg N, Sihvonen P, Kaila L. 2015. Elusive ditrysian phylogeny: an account of combining systematized morphology with molecular data (Lepidoptera). BMC Evol Biol. 15:260. 26589618 10.1186/s12862-015-0520-0 PMC4654798

[CIT0006] Kim JS, Kim MJ, Jeong JS, Kim I. 2018. Complete mitochondrial genome of *Saturnia jonasii* (Lepidoptera: Saturniidae): genomic comparisons and phylogenetic inference among Bombycoidea. Genomics. 110(5):274–282. 29191682 10.1016/j.ygeno.2017.11.004

[CIT0007] Kim JS, Park JS, Kim MJ, Kang PD, Kim SG, Jin BR, Han YS, Kim I. 2012. Complete nucleotide sequence and organization of the mitochondrial genome of eri-silkworm, *Samia Cynthia ricini* (Lepidoptera: Saturniidae). J Asia Pac Entomol. 15(1):162–173.

[CIT0008] Kim MI, Baek JY, Kim MJ, Jeong HC, Kim KG, Bae CH, Han YS, Jin BR, Kim I. 2009. Complete nucleotide sequence and organization of the mitogenome of the red-spotted apollo butterfly, *Parnassius bremeri* (Lepidoptera: Papilionidae) and comparison with other lepidopteran insects. Mole Cells. 28(4):347–363. 10.1007/s10059-009-0129-5 19823774

[CIT0009] Kim MJ, Jeong JS, Kim JS, Jeong SY, Kim I. 2017. Complete mitochondrial genome of the lappet moth, *Kunugia undans* (Lepidoptera: Lasiocampidae): genomic comparisons among macroheteroceran superfamilies. Genet Mol Biol. 40(3):717–723. 28767123 10.1590/1678-4685-GMB-2016-0298 PMC5596373

[CIT0010] Kim MJ, Wang AR, Park JS, Kim I. 2014. Complete mitochondrial genomes of five skippers (Lepidoptera: Hesperiidae) and phylogenetic reconstruction of Lepidoptera. Gene. 549(1):97–112. 25058696 10.1016/j.gene.2014.07.052

[CIT0011] Kim MJ, Wan X, Kim KG, Hwang JS, Kim I. 2010. Complete nucleotide sequence and organization of the mitochondrial genome of endangered *Eumenis autonoe* (Lepidoptera: Nymphalidae). Afr J Biotechnol. 9:735–754.

[CIT0012] Lanfear R, Calcott B, Ho SY, Guindon S. 2012. PartitionFinder: combined selection of partitioning schemes and substitution models for phylogenetic analyses. Mol Biol Evol. 29(6):1695–1701. 22319168 10.1093/molbev/mss020

[CIT0013] Lanfear R, Calcott B, Kainer D, Mayer C, Stamatakis A. 2014. Selecting optimal partitioning schemes for phylogenomic datasets. BMC Evol Biol. 14:82. 24742000 10.1186/1471-2148-14-82 PMC4012149

[CIT0014] Lanfear R, Frandsen PB, Wright AM, Senfeld T, Calcott B. 2016. PartitionFinder 2: new methods for selecting partitioned models of evolution for molecular and morphological phylogenetic analyses. Mol Biol E. 34:772–773. 10.1093/molbev/msw260 28013191

[CIT0015] Liu QN, Chai XY, Bian DD, Zhou CL, Tang BP. 2016. The complete mitochondrial genome of *Plodia interpunctella* (Lepidoptera: Pyralidae) and comparison with other Pyraloidea insects. Genome. 59(1):37–49. 26701149 10.1139/gen-2015-0079

[CIT0016] Miller MA, Pfeiffer W, Schwartz T. Creating the CIPRES science gateway for inference of large phylogenetic trees. Gateway Computing Environments Workshop (GCE). New Orleans (LA): 2010.

[CIT0017] Mutanen M, Wahlberg N, Kaila L. 2010. Comprehensive gene and taxon coverage elucidates radiation patterns in moths and butterflies. Proc Roy Soc B. 277(1695):2839–2848. 10.1098/rspb.2010.0392 PMC2981981 20444718

[CIT0018] Park JS, Kim MJ, Ahn SJ, Kim I. 2015. Complete mitochondrial genome of the grass moth *Glyphodes quadrimaculalis* (Lepidoptera: Crambidae). Mitochondrial DNA. 26(2):247–249. 24021007 10.3109/19401736.2013.823183

[CIT0026] Park JS, Kim MJ, Jeong SY, Kim SS., Kim I. 2016. Complete mitochondrial genomes of two gelechioids, Mesophleps albilinella and Dichomeris ustalella (Lepidoptera: Gelechiidae), with a description of gene rearrangement in Lepidoptera. Curr Genet. 62(4):809–826. 26952721 10.1007/s00294-016-0585-3

[CIT0019] Regier JC, Mitter C, Zwick A, Bazinet AL, Cummings MP, Kawahara AY, Sohn JC, Zwickl DJ, Cho S, Davis DR, et al. 2013. A large-scale, higher-level, molecular phylogenetic study of the insect order Lepidoptera (moths and butterflies). PLOS One. 8(3):e58568. 23554903 10.1371/journal.pone.0058568 PMC3595289

[CIT0020] Ronquist F, Teslenko M, Mark P, Ayres DL, Darling A, Höhna S, Larget B, Liu L, Suchard MA, Huelsenbeck JP. 2012. MrBayes 3.2: efficient Bayesian phylogenetic inference and model choice across a large model space. Syst Biol. 61(3):539–542. 22357727 10.1093/sysbio/sys029 PMC3329765

[CIT0021] Shin YH. 2001. Coloured illustrations of the moths of Korea. Seoul, Korea: Academybook Publishing Co. Ltd; p. 272.

[CIT0022] Stamatakis A. 2014. RAxML version 8: a tool for phylogenetic analysis and post-analysis of large phylogenies. Bioinformatics. 30(9):1312–1313. 24451623 10.1093/bioinformatics/btu033 PMC3998144

[CIT0023] Timmermans MJ, Lees DC, Simonsen TJ. 2014. Towards a mitogenomic phylogeny of Lepidoptera. Mol Phylogenet E. 79:169–178. 10.1016/j.ympev.2014.05.031 24910155

[CIT0024] Wan X, Kim MJ, Kim I. 2013. Description of new mitochondrial genomes (*Spodoptera litura*, Noctuoidea and *Cnaphalocrocis medinalis*, Pyraloidea) and phylogenetic reconstruction of Lepidoptera with the comment on optimization schemes. Mol Biol Rep. 40(11):6333–6349. 24057247 10.1007/s11033-013-2748-3

[CIT0025] Yang M, Song L, Shi Y, Li J, Zhang Y, Song N. 2019. The first mitochondrial genome of the family Epicopeiidae and higher-level phylogeny of Macroheterocera (Lepidoptera: Ditrysia). Int J Biol Macromol. 136:123–132. 31199977 10.1016/j.ijbiomac.2019.06.051

